# The impact of illness in patients with moderate to severe gastro-esophageal reflux disease

**DOI:** 10.1186/1471-230X-5-23

**Published:** 2005-07-10

**Authors:** Samer El-Dika, Gordon H Guyatt, David Armstrong, Alessio Degl'innocenti, Ingela Wiklund, Carlo A Fallone, Lisa Tanser, Sander Veldhuyzen van Zanten, Diane Heels-Ansdell, Peter Wahlqvist, Naoki Chiba, Alan N Barkun, Peggy Austin, Holger J Schünemann

**Affiliations:** 1 Division of Gastroenterology, Veterans affairs medical center, Salem, Virginia, USA; 2 Department of Clinical Epidemiology and Biostatistics' McMaster University, Hamilton, Ontario, Canada; 3 Department of Medicine, McMaster University, Hamilton, Ontario, Canada; 4 AstraZeneca R&D, Clinical Science, Mölndal, Sweden; 5 Division of Gastroenterology, McGill University Health Center, Montreal, Quebec, Canada; 6 AstraZeneca R&D, Canada, Mississauga, Ontario, Canada; 7 Division of Gastroenterology, Dalhousie University, Halifax, Nova Scotia, Canada; 8 Surrey GI Clinic/Research, Guelph, Ontario, Canada; 9 Department of Medicine, School of Medicine and Biomedical Sciences, University at Buffalo, State University of New York, Buffalo, New York, USA; 10 Division of Clinical Research Development and Information Translation/INFORMA, Italian National Cancer Institute, Rome/Istituto Regina Elena, Rome, Italy

## Abstract

**Background:**

Gastro-esophageal reflux disease (GERD) is a common disease. It impairs health related quality of life (HRQL). However, the impact on utility scores and work productivity in patients with moderate to severe GERD is not well known.

**Methods:**

We analyzed data from 217 patients with moderate to severe GERD (mean age 50, SD 13.7) across 17 Canadian centers. Patients completed three utility instruments – the standard gamble (SG), the feeling thermometer (FT), and the Health Utilities Index 3 (HUI 3) – and several HRQL instruments, including Quality of Life in Reflux and Dyspepsia (QOLRAD) and the Medical Outcomes Short Form-36 (SF-36). All patients received a proton pump inhibitor, esomeprazole 40 mg daily, for four to six weeks.

**Results:**

The mean scores on a scale from 0 (dead) to 1 (full health) obtained for the FT, SG, and HUI 3 were 0.67 (95% CI, 0.64 to 0.70), 0.76 (95% CI, 0.75 to 0.80), and 0.80 (95% CI, 0.77 to 0.82) respectively. The mean scores on the SF-36 were lower than the previously reported Canadian and US general population mean scores and work productivity was impaired.

**Conclusion:**

GERD has significant impact on utility scores, HRQL, and work productivity in patients with moderate to severe disease. Furthermore, the FT and HUI 3 provide more valid measurements of HRQL in GERD than the SG. After treatment with esomeprazole, patients showed improved HRQL.

## Background

The global prevalence of gastro-esophageal reflux disease (GERD), defined as heartburn once daily, is estimated to range from 5 to 7% but varies widely and depends on the definition used [1]. For example, 25% of the adult population in Belgium [2], nearly 18% in Australia [3], 20% in the United States and 9 % in Canada reported GERD symptoms once a week or more [4,5]. While heartburn is the leading symptom in GERD, the disease is associated with a broad range of esophageal problems, including acid regurgitation, epigastric pain, esophageal erosions and complications such as Barrett's esophagus, esophageal adenocarcinoma and esophageal stricture. GERD is also associated with a variety of extra-esophageal problems, including sleep disturbances, noncardiac chest pain, asthma, chronic cough and hoarseness [6].

Clinicians and health services researchers are becoming more aware of the importance of patient-reported outcomes (PROs), including health related quality of life (HRQL) in understanding the burden of disease and the outcome of medical treatment. While physiologic measures provide information to clinicians, these outcomes are often not important to patients [7] and they correlate poorly with functional status or well-being, the areas in which patients are mostly interested. For example, the majority of patients with typical symptoms of GERD do not have endoscopic evidence of esophagitis [8]. PROs assist in providing a better understanding of treatment outcomes from the patient's perspective by translating clinical improvement into patient-important outcomes. Moreover, HRQL assessment is important for measuring quality of care, clinical effectiveness, and in reimbursement decisions [9,10].

The use of validated questionnaires is appropriate for measuring PROs in clinical trials [11,12]. There are two categories of HRQL measures; disease-specific and generic HRQL instruments [13,14]. Disease-specific instruments are used to describe the burden of disease and treatment outcomes in patients with a specific disease, and generic instruments measure the overall HRQL of patients, including physical, emotional, and social function, as well as their level of general performance at work and in daily life across different diseases [15].

Utility measures, one type of generic health status measures, are based on economic and decision theory [16]. These instruments measure patient preferences and generate preference or utility scores for respondents' health states on a 0 to 1.0 scale where 0 typically equals dead and 1.0 is full health [17]. In this manuscript we will use the term "utility" for all scores generated with preference based instruments although. The standard gamble (SG) is regarded as the reference standard for utility measurement [18-20]. Another utility instrument is the feeling thermometer (FT), a visual analogue scale presented in the form of a thermometer [18]. When completing this instrument, patients choose the score on the thermometer that represents the value they place on their health state. It is far simpler than the SG and has shown good responsiveness and validity in several studies [21-24]. The health utility index 3 (HUI 3) is a multi attribute utility instrument designed to classify a patient's health status based on rating of a set of defined items [25,26].

The aim of this analysis was to address the impact of GERD on utility scores, HRQL and work productivity in patients with moderate to severe GERD and to evaluate the construct validity of utility instruments as measures of HRQL.

## Methods

### Patients

We enrolled 249 uninvestigated outpatients with a clinical diagnosis of moderate to severe GERD in 13 specialty centers and 4 general practices across Canada between March 2002 and March 2003. Table 1 lists the definitions of symptoms severity. To evaluate the aims described in the introduction of this manuscript, we utilized data from a study that had as primary aim the comparison of two different formats of administering the SG and the FT [27]. Therefore, the current publication describes the data from the baseline visit of the 217 patients (87%) who completed the study and the responsiveness of the QOLRAD and the four symptoms questionnaire after four to six weeks of treatment with a proton pump inhibitor (PPI), esomeprazole. The main inclusion criteria were a clinical diagnosis of GERD, main symptom of heartburn, age greater 18 years, symptomatic for three months or longer, and off PPI for the 2 weeks prior to questionnaire administration. GERD was defined as a burning feeling, rising from the stomach or lower part of the chest up towards the neck. We describe the detailed eligibility criteria elsewhere [27].

### Study design

Patients completed several utility and HRQL instruments during a clinic visit and provided demographic and clinical data. The visit lasted approximately 80 minutes. An experienced research coordinator from the method center trained all site interviewers in a daylong session in HRQL instrument administration. Ethic review boards at all study sites approved the study protocol and all patients signed an informed consent form prior to enrollment in the study.

### Utility measures

The feeling thermometer (FT) is a visual analogue scale shown as a thermometer in which the best state is full health (equal to a score of 100) and the worst state is dead (a score of 0) [18]. It has shown good responsiveness and validity in several studies [21-24]. In this trial, we used a self-administered form of the FT [27,28].

The SG [18-20] offers patients two options from which a choice must be made: Choice A is the certain outcome that the patient will stay in a health state (their own health state, or a marker state) for t years until death. We varied t depending on the patient's age as follows: patients aged more than 80 years, t = the rest of the patient's lifetime; age 76 – 80 years, t = 10 years; age 66 – 75 years, t = 15 years; age 56 – 65, t = 25 years; age 46 – 55 years, t = 30 years; age 36 – 45 years, t = 35 years; age 26 – 35 years, t = 40 years; age 18 – 25 years, t = 45 years. Specifying the duration of remaining life means that patients use the same time frame as other patients of the same age, and reduces the random error that might result from patients inferring different time frames. Varying time frame by age minimizes an additional lack of realism that could arise if one chose a single time frame and either young patients have an unrealistically short duration of remaining life, or old patients have an unrealistically long duration. The alternative (choice B) is a hypothetical treatment with 2 outcomes: 1) returning to full health (probability p) for t years, at the end of which the patient dies or 2) immediate death (probability 1-p). Interviewers used a chance board with a ping-pong approach varying the probability p in steps of 0.05 to obtain the value, p*, where the patient considered choice A equal to choice B. This indifference probability, p*, is the utility value for the patient's own health in choice A in the interval from dead (= 0) to full health (= 1). The greater the respondent's willingness to accept the risk of a worse outcome (e.g dead) to avoid the health state in choice A, then the lower is the utility of the state in choice A to them.

The HUI 3 is a 15 item self-administered questionnaire. It has 8 attributes that include vision, hearing, speech, ambulation, dexterity, emotion, cognition and pain. We calculated a utility score on a 0 to 1.0 scale where 0 represents dead and 1.0 represents full health [26]. HUI has been shown to be a reliable, responsive and valid measure in a wide variety of clinical studies [25].

In addition we used the four symptoms questionnaire that comprises a series of four questions on which patients rate how they felt for the past week using a seven-point Likert scale ranging from no problem to very severe problem. The four symptoms questionnaire evaluates heartburn, acid reflux, belching, and stomach ache.

### Disease-specific HRQL

The quality of life in reflux and dyspepsia (QOLRAD) consists of 25 items across five dimensions: emotional distress, sleep dysfunction, vitality, food/drink problems, and physical/social functioning. Patients provide answers on a seven-point Likert-type scale. The lower the value, the more severe is the impact on daily functioning. The QOLRAD is reliable, valid and responsive [29-31].

### GERD-specific work productivity and activity impairment questionnaire

The GERD-specific Work Productivity and Activity Impairment questionnaire (WPAI-GERD) contains 8 items (Table 2) that uses a one-week recall period and measures absence from work, reduced productivity while at work and reduced productivity while doing regular daily activities other than work [32].

### Health profile measure

The Medical Outcomes Study Short Form-36 (SF-36) is a generic instrument that assesses a wide range of health problems, including GERD [34]. It consists of 8 domains including physical functioning, role limitations-physical, bodily pain, general health, vitality, social functioning, role limitations-emotional, and mental health. The SF-36 scores range from 0 to 100, with higher scores indicating better functioning and well-being.

### HRQL and utilities for other health states

We compared the SF-36 scores for the Canadian general population reported by Hopman et al. [33], and the US general population, depression, hypertension, diabetes extracted from the Medical Outcome Study and reported by Revicki et al. [34-37] to the SF-36 scores of our study patients. In addition, we compared the utility scores of patients enrolled in this study with those of other patients reported previously, as utility ratings are comparable across conditions because they provide scores between 0 (dead) and 1 (full health) that are not disease specific [38,39]. A priori, we determined that we would focus on patients with common diseases for whom utility assessments are available.

### Statistical analysis

We report baseline instrument scores as means with 95% confidence intervals (CI). The QOLRAD and the four symptoms questionnaire responsiveness to treatment are shown as mean change scores with 95% CI. We also evaluated the cross-sectional construct validity of the utility instruments by calculating Pearson's correlation coefficients of the scores on the FT, SG and HUI 3 with the validation instruments the QOLRAD, the SF-36, and the four symptoms questionnaire. We assumed that higher correlations with the validation instruments would indicate greater construct validity. For interpretation of the correlations we considered correlations of less than 0.2 as very weak, from 0.2 to 0.35 as weak, from 0.35 to 0.5 as moderate and of more than 0.5 as strong. We compared mean physical and mental component summary scores on the SF-36 of our study population and the previously reported scores of the Canadian population, the US population, clinical depression, diabetes, and hypertension using Student's t tests [33-37].

## Results

### Baseline characteristics and burden of disease

Table 3 shows the baseline characteristics of the patients. The mean age was 50 years (range 20 to 82), 53% were females, 69%% were employed, and the mean number of months since diagnosis was 86 (range 1 to 504). Table 4 presents mean QOLRAD scores and the mean scores for the four symptoms questionnaire along with the mean scores change after 4 weeks of PPI treatment. GERD has the greatest impact on the vitality and food/drink domains of the QOLRAD (scores of 4.3 and 3.8 respectively). The most severe symptoms were heartburn and acid reflux with scores of 4.5 and 4.1 respectively. PPI treatment significantly improved the scores of the QOLRAD and the four symptoms.

### WPAI-GERD

A total of 153 (71%) patients were employed. The percentage of overall work impairment secondary to GERD that included absence from work plus time lost due to reduced productivity was 16% (95% CI, 12.9 to 18.8), which corresponds to 6.7 hours lost per week due to GERD symptoms. Furthermore, the reduced productivity during activities other than work in 216 patients was 21% (95% CI, 18.0 to 24.0).

### Construct validity of the utility instruments

The correlations of the FT and HUI 3 with the QOLRAD and SF-36 domains were moderate. However, the SG showed lower correlations than the FT and the HUI 3. The FT had the highest, albeit weak correlation with the four symptoms questionnaire (Table 5).

### Comparison of utility scores with other diseases

The systematic review by Morimoto et al. of utility measures reported SG weighted means for asthma, chronic renal failure, and angina pectoris of 0.88 (range 0.82–0.91), 0.52 (range 0.49–0.55), and 0.76 (range 0.64–0.97), respectively [38]. The systematic review by Post et al. revealed time trade off (TTO) and SG in survivors of minor stroke to be 0.72 (range 0.71–0.81), and 0.89 (range 0.81–0.95) respectively [39]. We previously reported baseline scores for the FT and SG in patients with moderate to severe COPD to be 0.60 (SD 0.18), and 0.66 (SD 0.27) respectively [24]. In this study, the utilities obtained for the FT, SG, and HUI 3 were 0.67 (95% CI, 0.64–0.70), 0.78 (95% CI, 0.75–0.80), and 0.80 (95% CI, 0.77–0.82) respectively.

### Comparison of the SF-36 scores to other diseases and the general population

The mean scores on the SF-36 range from 42.8 to 47.7 across the different SF-36 domains (Table 6). These scores are significantly lower than the Canadian and US general population mean scores on the physical and mental component summaries (table 7) [33,35]. Table 7 also demonstrates the comparison of the SF-36 scores of our patients to other groups of patients included in the Medical Outcomes Study and reported by Revicki et al. [35,36,40].

## Discussion

We determined the impact of GERD on utility scores, HRQL and work productivity in patients with moderate to severe GERD and the cross-sectional construct validity of three utility instruments (FT, SG, and HUI 3). Although the comparisons we made are indirect, the results of this study indicate that GERD causes important reductions in HRQL and utility when compared to the Canadian and US general population and to those of patients with a range of other chronic conditions. Heartburn and acid reflux were reported by our patients to have the worst impact on symptoms using the four symptoms questionnaire. The QOLRAD as well as the symptom scores improved after esomeprazole treatment although this study did not include a placebo group. In addition, our data indicate that GERD causes a considerable loss in work productivity.

The strengths of this study include the use of several utility and HRQL instruments that allow comparison to other chronic conditions. By using several quality of life instruments, we have studied GERD patients more comprehensively than previous studies [31,32,35]; however, our results are confined to GERD patients with moderate to severe symptoms who were participating in a clinical study. In addition, our comparison with population data is based on historical data. Despite the impressive responsive of the QOLRAD scores to esomeprazole treatment, one has to keep in mind that this study was not a randomized controlled trial. In regards to evaluation of validity, another limitation of our study is that we did not generate a priori predictions regarding correlations between the utility instruments and other measures. Had we generated such a priori predictions our conclusion about the validity of the instruments might be stronger.

Data on utility measures in GERD patients are sparse. We observed important disutility measured with the FT, SG, and HUI 3. The results suggest that the FT and HUI 3 are valid tools for the assessment of HRQL in patients with GERD. We were specifically interested in exploring the relative validity of utility instruments in patients with GERD.

In general, the correlations with other HRQL instruments were moderate. In contrast, the SG showed poor construct validity. Moreover, the FT shows better correlation with the four symptoms questionnaire than the SG and the HUI 3. Thus, our findings suggest that the FT and the HUI 3 are more appropriate indicators of HRQL impairment than the SG in these patients. Studies that use the FT, SG, HUI and SF-36 simultaneously are rare. We have previously observed a similar pattern of correlations in patients with chronic obstructive pulmonary disease (COPD) [24]. The correlations of the FT and HUI 3 with the SF-36 were higher compared to those of the SG with the SF-36. Thus, there is external evidence that the FT and HUI 3 show greater validity for the assessment of HRQL than the SG.

The data also indicate that the disutility in patients with moderate to severe GERD is similar to that of moderate to severe COPD [24]. The utility scores obtained with the SG are lower than what was previously reported for SG scores in patients with asthma [38] and comparable to those of minor stroke survivors [39].

We also found reductions in the SF-36 scores on all 8 domains in GERD patients. Compared to the Canadian and US general population, patients in this study had significantly reduced scores in the SF-36 MCS and PCS [33-36]. The SF-36 PCS scores are comparable to patients with clinical depression and hypertension. On the other hand, GERD patients have significantly worse SF-36 MCS scores than patients with diabetes mellitus and depression.

The QOLRAD results suggest that GERD has the greatest impact on the vitality and food/drink domains. These results are similar to those reported by Wiklund et al. [31] confirming the negative impact of GERD on the daily functioning of affected patients.

Health administrators and payers are interested in the magnitude of work productivity loss due to GERD [41]. Wahlqvist et al. showed that patients with GERD symptoms report 23% reduced productivity while at work, and 30% reduced productivity while doing regular daily activities in a Swedish population [32]. Our study supports the finding of impaired productivity in patients with moderate to severe GERD, but the estimates of work loss are somewhat lower demonstrating that about 16% of the work time is lost due to the illness. Since GERD affects approximately 9% of the Canadian population [5], impaired productivity has important economic consequences on society if it is not treated effectively.

## Conclusion

In summary, we found that GERD has significant impact on utilities scores, HRQL and work productivity in patients with moderate to severe illness. The impact of the disease is similar in magnitude to other chronic conditions that are less responsive to treatment. In addition, utility instruments such as the FT and HUI 3 provide valid measurements of the impact of GERD on HRQL.

## Competing interests

This study was funded by a grant from AstraZeneca, Sweden. Several authors of this manuscript (ADI, IW, LT, PW) are employees of the sponsor. Several authors have received research funding and/or honoraria from the sponsor (HJS, GHG, DA, NC, AB, CF, SVZ). NC and SVZ received honoraria for speaking about related conditions such as GERD. SVZ is a member of an AstraZeneca advisory board. AB is a consultant to AstraZeneca, Canada. The honoraria of Dr. Holger Schünemann and Dr. Gordon Guyatt were deposited into research accounts at the University at Buffalo and McMaster University.

## Authors' contributions

Samer El-Dika was the project leader for this manuscript interpreted the data, performed data analysis and drafted the final version of the manuscript as well as early versions. Gordon H Guyatt and Holger Schünemann were the principal investigators of the study, wrote the clinical protocol and grant application, are responsible for the study protocol, interpreted data and participated in writing the final as well as early versions of this manuscript. Ingela Wiklund contributed to the study protocol, interpreted the data and edited the manuscript. Diane Heels-Ansdell was responsible for the statistical analysis and edited the final manuscript as well as early versions. David Armstrong was co-principal investigator of the study, revised the clinical protocol, assessed patients, interpreted data and edited the final manuscript as well as early versions. Peter Wahlqvist interpreted the WPAI-GERD data and edited the manuscript. Carlo A Fallone, Naoki Chiba and Sander Veldhuyzen van Zanten revised the clinical protocol, assessed patients, interpreted data and edited the final manuscript as well as early versions. Alessio Degli'Innocenti, Alan N Barkun, and Peggy Austin revised the clinical protocol, interpreted data and edited the final manuscript as well as early versions. Peggy Austin also co-ordinated the study. Lisa Tanser contributed to co-ordination of the study. All authors approved the final manuscript. The AstraZeneca global publications group reviewed the manuscript and made minor wording suggestions to clarify methodological aspects.

## Pre-publication history

The pre-publication history for this paper can be accessed here:



## Figures and Tables

**Table 1:** Definition of symptom severity

Moderate problem:	Cannot be ignored but does not influence my daily life.
Moderately severe problem:	Cannot be ignored and occasionally limits my daily activities.
Severe problem:	Cannot be ignored and often limits my concentration on daily activities.
Very severe problem:	Cannot be ignored and markedly limits my daily activities and often requires rest.

**Table 2:** GERD-specific work productivity and activity impairment questionnaire (WPAI-GERD)

1. Are you currently employed?
2. During the past 7 days, how many days did you work?
3. How many of those days did you have reflux symptoms while working?
4. During the past 7 days, how many hours were you absent from work because of problems associated with your reflux symptoms?
5. During the past 7 days, how many hours were you absent from work for any other reason than problems associated with your reflux symptoms?
6. During the past 7 days, how many hours did you actually work?
7. During the past 7 days, how much did your reflux symptoms on average affect your normal productivity while you were working?
8. During the past 7 days, how much did your reflux symptoms on average affect your normal productivity while you were doing your regular activities, other than working?

**Table 3:** Baseline characteristics of patients with moderate or severe GERD mean age 50 years, range 20 to 82

	Frequency (Percentage) (N 217)
Female	114 (52.5)
Single	33 (15.2)
Lives alone	23 (10.6)
Employed	153 (71.0)
Never smoked	94 (43.5)
Ever smoked	122 (56.5)
Caucasian	191 (88.0)
Other ethnic groups	26 (12.0)
Moderate GERD*	112 (51.6)
Moderately severe GERD*	74 (34.1)
Severe GERD*	27 (12.5)
Very severe GERD*	4 (1.8)

*GERD: Gastroesophageal reflux disease

**Table 4:** QOLRAD and four symptoms questionnaire baseline scores and mean change scores after 4 week treatment with PPI

Instrument	Mean baseline score (95% CI)	Mean change score (95% CI)
QOLRAD emotional Distress	4.5 (4.3, 4.7) (N = 217)	2.0 (1.9, 2.2) (N = 217)
QOLRAD sleep Disturbance	4.5 (4.3, 4.7) (N = 217)	2.1 (1.9, 2.3) (N = 217)
QOLRAD food/drink problems	3.8 (3.7, 4.0) (N = 217)	2.5 (2.3, 2.6) (N = 217)
QOLRAD physical/social	5.5 (5.3, 5.6) (N = 217)	1.3 (1.1, 1.4) (N = 217)
QOLRAD vitality	4.3 (4.1, 4.5) (N = 217)	2.1 (1.9, 2.3) (N = 217)
Stomach pain	3.9 (3.7, 4.1) (N = 217)	-1.9 (-2.2, -1.7) (N = 217)
Heartburn	4.5 (4.3, 4.7) (N = 217)	-2.9 (-3.1, -2.7) (N = 216)
Belching	3.6 (3.4, 3.8) (N = 216)	-1.6 (-1.8, -1.4) (N = 216)
Acid reflux	4.1 (3.9, 4.3) (N = 215)	-2.4 (-2.6, -2.2) (N = 212)

QOLRAD: Quality of life in reflux and dyspepsia, PPI: Proton pump inhibitor

**Table 5:** Correlation of the FT, SG and HUI 3 with the validation instruments (P-values)

Instrument	FT	SG	HUI 3
SF-36 PCS (N = 212)	0.40 (<0.001)	0.31 (<0.001)	0.46 (<0.001)
SF-36 MCS (N = 212)	0.34 (<0.001)	0.22 (<0.001)	0.54 (<0.001)
QOLRAD emotional Distress	0.48 (<0.001)	0.27 (<0.001)	0.38 (<0.001)
QOLRAD sleep disturbance	0.39 (<0.001)	0.25 (<0.001)	0.23 (<0.001)
QOLRAD food/drink problem	0.37 (<0.001)	0.28 (<0.001)	0.25 (<0.001)
QOLRAD physical/social	0.38 (<0.001)	0.26 (<0.001)	0.41 (<0.001)
QOLRAD vitality	0.48 (<0.001)	0.30 (<0.001)	0.44 (<0.001)
Stomach pain	0.32 (<0.001)	0.15 (0.032)	0.26 (<0.001)
Heartburn	0.32 (<0.001)	0.13 (0.059)	0.09 (0.174)
Belching (N = 216)	0.20 (0.003)	-0.09 (0.193)	0.09 (0.189)
Acid reflux (N = 215)	0.34 (<0.001)	0.15 (0.015)	0.14 (0.046)

FT: Feeling thermometer; SG: Standard gamble; HUI 3: Health utility index 3; SF-36: Short form 36; PCS: Physical component summary; MCS: Mental component summary; QOLRAD: Quality of life in reflux and dyspepsia. N = 217 unless indicated otherwise.

**Table 6:** SF-36 mean scores

Instrument	Mean	95% CI
SF-36 physical functioning	46.6 (N = 213)	45.4, 47.8
SF-36 physical-Role	45.5 (N = 216)	44.0, 47.0
SF-36 bodily pain	42.8 (N = 217)	41.6, 44.1
SF-36 general health	46.2 (N = 216)	44.9, 47.5
SF-36 vitality	45.9 (N = 217)	44.6, 47.3
SF-36 social functioning	47.7 (N = 217)	46.3, 49.1
SF-36 role emotional	46.5 (N = 216)	44.9, 48.1
SF-36 mental health	46.9 (N = 217)	45.5, 48.3

SF-36: Short form 36

**Table 7:** Baseline mean (standard deviation) SF-36 physical component summary (PCS) and mental component summary (MCS) scores for patients with gastroesophageal reflux disease compared with data reported by Revicki et al. [35] and Hopman et al. [33]

SF-36 scale	GERD (this study)	Canadian population§ (N = 9,423)	US Population† (N = 2,474)	Clinical depression† (N = 502)	Diabetes† (N = 541)	Hypertension† (N = 2,089)
PCS	45.1 (n = 212) (8.7)	51.4* (8.5)	50.0* (10.0)	45.0 (12.1)	41.5* (11.3)	44.3 (10.8)
MCS	47.6 (n = 212) (11.0)	52.6* (8.5)	50.0* (10.0)	34.8* (12.2)	51.9* (9.6)	52.2* (9.3)

*P < 0.001 compared to our GERD patients.

§ Data from Hopman et al.

† Data from Revicki et al.
